# From active play to sedentary lifestyles: understanding the decline in physical activity from childhood through adolescence—a systematic review

**DOI:** 10.3389/fpubh.2025.1636891

**Published:** 2025-11-06

**Authors:** Jean de Dieu Habyarimana, Etienne Tugirumukiza, He Sun, Amith Pathirana, Ke Zhou

**Affiliations:** 1 Department of Physical Education, Henan University, Kaifeng, China; 2 Capital University of Physical Education and Sports, Beijing, China

**Keywords:** youth physical inactivity, health outcome, exercise promotion, adolescents, systematic review

## Abstract

The decline in physical activity (PA) from childhood through adolescence is an escalating global concern with far-reaching implications for health and wellbeing. While prior research has examined general PA trends, critical gaps remain regarding the precise onset of decline, contributing factors, and the most effective intervention strategies. This systematic review synthesizes evidence on: (a) the age or developmental stage at which PA levels significantly decline, (b) key factors influencing this decline, and (c) interventions shown to be effective in slowing or preventing it. A comprehensive search was conducted across four electronic databases: Scopus, Web of Science, Google Scholar, and CORE guided by the Preferred Reporting Items for Systematic Reviews and Meta-Analyses (PRISMA) framework. From an initial pool of 14,621 records, 34 studies met the inclusion criteria. Findings reveal that PA levels begin to decline as early as age 7, with the most substantial drop occurring around age 9. Modifiable factors such as self-efficacy, motivation, screen time, and academic workload emerged as key contributors to this trend. Among intervention strategies, school-based, multicomponent, and theory-driven approaches, particularly those incorporating autonomy-supportive teaching, addressing both PA and sedentary behaviors, and engaging multiple stakeholders, demonstrated the strongest effectiveness. These results underscore the urgent need for early, multidimensional interventions to sustain PA engagement across developmental stages. Stakeholders including schools, policymakers, and researchers should prioritize integrated PA promotion strategies to reverse early inactivity trends. Future research should focus on the long-term sustainability of these interventions beyond the school context to ensure enduring behavioral change.

## 1 Introduction

Physical activity (PA) is far more than a means of energy expenditure, it's a powerful catalyst for children's holistic development. From building stronger bodies to fostering emotional resilience and social competence, its benefits are profound and well-documented. Numerous studies have highlighted its protective role against obesity, cardiovascular diseases, and mental health conditions such as anxiety and depression (1–5). Recognizing this, leading global health bodies have issued clear mandates: sustained engagement in PA from early childhood through adolescence is essential to cultivate lifelong habits of health and wellbeing (2, 6). Despite these calls to action, a steady and alarming decline in PA levels is being documented across childhood and adolescence. This trend, now widely recognized, carries serious long-term implications, not just for individual health, but for entire public health systems (7, 8).

Crucially, diminished PA during these formative years has been linked to a broad spectrum of negative outcomes. Physically, it heightens the risk of obesity, metabolic disorders, and cardiovascular complications (7, 9–12). Psychologically, its absence undermines emotional stability, leaving youth more vulnerable to stress, anxiety, and depressive symptoms (13–15, 104). Cognitive functioning also suffers. Recent research increasingly shows that regular PA enhances memory, attention span, and executive functioning, tools vital for academic achievement (16–19). In parallel, physical inactivity is known to impede motor skill development, coordination, and physical fitness, all critical not only for health but for building confidence and social bonds (20, 21). Taken together, these effects point to one undeniable truth: the decline in PA during youth is a multi-dimensional threat.

Interestingly, while prior studies have long identified adolescence as a turning point for decreased PA (18, 22), newer findings complicate this narrative. Contrary to earlier assumptions that PA remains stable throughout childhood and then plunges in adolescence (23, 24), emerging evidence suggests a more insidious pattern, one that begins much earlier, during late childhood (25–30).

If the decline initiates earlier than previously thought, then current interventions, many of which target adolescents may already be arriving too late. Prevention strategies must be reimagined. Compounding this challenge is the web of contributing factors: social norms, psychological barriers, environmental limitations, institutional policies, even biological predispositions all implicated to varying degrees (31–33). However, how these forces intersect and amplify one another remains poorly understood. This complexity underscores the need for a systems thinking approach, a holistic perspective that accounts for the interplay of individual, social, institutional, and environmental influences on young people's PA. By framing the issue within this interconnected context, we acknowledge that no single factor acts in isolation, and efforts to counteract declining activity must address these influences collectively.

Specifically, this systematic review aims to (1) pinpoint the precise age or developmental stage at which PA levels begin to meaningfully decline; (2) identify and synthesize the principal factors driving this decline; and (3) examine the evidence for interventions that have proven effective in mitigating or reversing this trend. While the methods and results sections respond to the first two objectives through rigorous literature analysis, the third is approached through integrative synthesis in the discussion section. In doing so, this review offers more than just a summary of evidence, it delivers an urgent call to action.

At a time when global campaigns to increase youth PA continue to face resistance and limited success (2, 34, 35), our findings offer clarity and direction. By identifying the when, why, and how of PA decline, this study equips educators, policymakers, and researchers with the insights needed to craft sustainable, multi-layered strategies that intervene early, before habits crystallize and activity gives way to inertia.

## 2 Material and methods

### 2.1 Protocol and registration

This review adhered to the Preferred Reporting Items for Systematic Reviews and Meta-Analyses (PRISMA) 2020 guidelines (36). The protocol was registered with the International Prospective Register of Systematic Reviews (PROSPERO) under registration number CRD42024613625, ensuring methodological transparency and alignment with predefined objectives.

### 2.2 Eligibility criteria and study selection

The eligibility criteria for study inclusion in this systematic review were based on the PICOS framework (101). Specifically: (a) Participants: Studies focusing on children and adolescents, both boys and girls aged 6–18 years, enrolled in elementary and secondary schools with prescribed physical education; (b) Intervention: Studies that utilized physical education (PE) classes or extra-curricular activities within school settings as the intervention medium; (c) Comparison: Children categorized in the age group immediately preceding the intervention group (6–18 years old) i.e., younger vs. older children; (d) Outcomes: Studies reporting on PA levels, including maintenance, decrease, or increase in PA and the associated factors; and (e) Study Design: Longitudinal studies, follow-up, prospective, cohort were included to capture changes in PA over time. These criteria ensured a focused and consistent approach to study selection, allowing for a comprehensive examination of the effects of PE interventions on PA patterns across childhood and adolescence.

### 2.3 Search strategy

**Figure 1:**
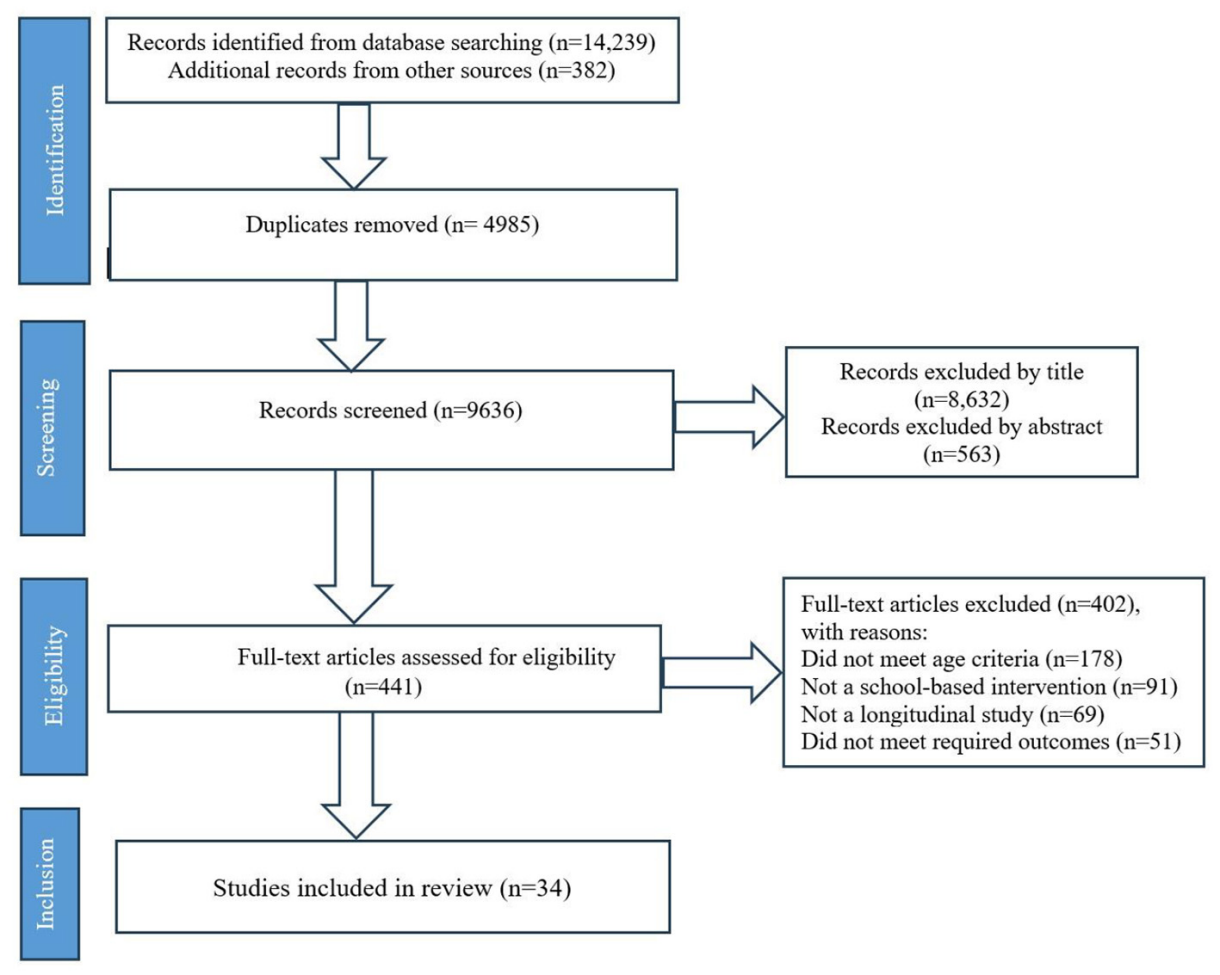
PRISMA Flow diagram outlining the study selection process.

A comprehensive literature search was conducted in Scopus, Web of Science, Google Scholar, and CORE, covering studies published between January 1, 1990, and November 30, 2024. Figure 1 shows the study selection process. Searches were conducted between November 1 and December 29, 2024. Only peer-reviewed articles published in English were included.

Search terms targeted the title, abstract, and keywords, and were combined using Boolean operators, including: (“Play” OR “Recreation” OR “Physical play”) AND (“Sedentary” OR “Inactivity” OR “Inactive lifestyle” OR “Screen time”) AND (“Physical activity” OR “Exercise” OR “Physical fitness” OR “Active lifestyle”) AND (“Decline” OR “Reduction” OR “Decrease” OR “Diminish” OR “Change” OR “Maintenance” OR “Tracking” OR “Variability” OR “Trend” OR “Trajectory”) AND (“Childhood” OR “Youth” OR “Pediatric” OR “Pre-adolescence” OR “Early adolescence” OR “Adolescence” OR “Teenager”) AND (“Intervention” OR “Prevention” OR “Promotion” OR “Program” OR “Policy” OR “Strategy” OR “Implementation”) AND (“Individual factors” OR “Social factors” OR “Environmental factors” OR “Biological factors” OR “Physiological factors” OR “Psychological factors”). To ensure subject relevance, the search was refined to include studies related to sports pedagogy, social science, recreation, PE, and youth health. To supplement database searches, bi-directional citation screening (37) was used to identify additional relevant studies. This included screening both references cited within selected studies and newer publications citing them.

### 2.4 Data collection process

All search results, including articles from citation screening, were imported into Covidence™, a systematic review management software (38), where duplicate entries were removed. Three independent reviewers, all with expertise in field of study related to physical activity and health among young people, conducted a blinded screening of titles and abstracts to assess eligibility. Discrepancies were resolved through discussion, with the lead author serving as an adjudicator when necessary. Articles passing initial screening underwent full-text review based on the predefined inclusion criteria.

The following data were systematically extracted from each included study: (1) Study Information: Author names, year of publication, and study location; (2) Participant Characteristics: Sample size, age range, and gender distribution; (3) Intervention details: Description of physical education (PE) interventions, including duration and frequency; (4) Comparison Groups: Age-based or gender-based comparisons; (5) Outcome Measures: Physical activity levels, including baseline and post-intervention data, and methods of assessment (e.g., self-report, accelerometer, observation); (6) Study Design and Methodology: Study type (longitudinal, cross-sectional, or randomized controlled trial) and duration of follow-up for longitudinal studies; (7) Findings: Changes in physical activity levels (decline, maintenance, or increase) across age groups, any differences observed between boys and girls and potential factors associated with such a change. Table 1 summarizes the characteristics and findings of the included studies.

**Table 1:** Synthesis of the articles included in the review.

**No**	**Study information**	**Participants**	**Intervention details**	**Comparison group**	**Outcome measures**	**Study design**	**Main findings**
1	Farooq et al. (25); England	545 individuals, newborn-15 years old	Light intensity PA combined with MVPA	Reference group	Actigraph GT1M accelerometer,	Longitudinal cohort, 8 years follow-up,	All trajectories declined from age 7 years
2	Farooq et al. (51); England	671 subjects, new born to 15 years, 49.6% boys & 50.4% girls	Light intensity & MVPA	Reference group	Actigraph GT1M accelerometer	Longitudinal cohort, 8 years follow-up,	Modifiable (active commuting to school, sports club membership, safe environment for outdoor play) and non-modifiable (sex, Socio-Economic Status SES) factors
3	McMurray et al. (40); USA	1,064 participants, 535 boys and 529 girls	PAs	Gender and ethnicity	Questionnaire and ergometer	Longitudinal, 7 years follow-up	PA s decline during Puberty (9–13years)
4	Corder et al. (24); UK	480 participants, 9–15years	LPA and MVPA	Gender, home location and bodyweight status	questionnaires and fit accelerometers	Longitudinal cohort study, 4 years follow-up	PAs decline during transition to adolescence 10–11 years
5	Azevedo et al. (95); Brazil	3,736 subjects, birth-18 years old	Leisure time PA, MVPA	Gender based	Interview, Questionnaire	Longitudinal study, 18 years follow-up	Decline of PAs started at the age of 11
6	Brodersen et al. (96); UK	5,863 students, 11–12 years old	VPA	Gender, racial and SES based	Questionnaire	Longitudinal study, 5 years follow-up	Decline of PAs decline starting from Age 11
7	Kimm et al. (47); USA	2,379 girls, 1,213 black and 1,166 white girls, 9/10–18/19 years	Overall PAs	Racial based	A 3-d diary (AD) and a habitual pattern questionnaire (HAQ)	Longitudinal study, 9 years follow-up	Overall PAs dramatically decline during transition from childhood to adolescence (10–11 years old)
**8**	Sember et al. (18); Slovenia	100 participants, 50 boys and 50 girls, 11–14 years old,	MVPA	representative sample of Slovenian schoolchildren (621)	Bodymedia SenseWear Pro Armband monitor	Cohort study	PAS (MVPA) decreased more than one quarter (34.96 min) during early adolescence (from age 11 to age 14) Environmental factors (reduction of PE classes and increment of schoolwork)
9	Ortega et al. (41); Estonia	1,800 subjects, 9–15 years old, 811 boys and 989 girls	MVPA and sedentary time	Gender	Actigraph accelerometers,	A cohort study, 6–9/10 years follow-up	The decline in MVPA and increase sedentary time were observed during preadolescence (9–12yearsold) larger in boys than girls.
10	Rangul et al. (97); Norway	2,348 subjects, 1,089 boys and 1,259, 13 years	MVPA	Gender	questionnaire	Longitudinal study, 4 years follow-up.	overweight, dissatisfied with life, and not actively participating in sports for boys and smoking, drinking, low maternal education, and physical inactivity for girls.
11	Schwarzfischer et al. (54); Germany	600 children, 8th week after birth,	LPA and MVPA	Gender	The SenseWear Armband	Longitudinal cohort, 5 years follow-up	The decline PAs started well during preadolescence period (8–11 years old); Influencing: factors were sex, country, and body size.
12	Husøy et al. (42); Norway	731 subjects, 9–15 years, 358 girls and 373 boys	PAs except water-based activities	Age	ActiGraph accelerometers	Longitudinal study, 6 yeas follow-up	The most prominent decline started from ages of 9–15 years.
13	Metcalf et al. (43); UK	300 children, 5–15 years,	Light and MVPAs	Gender	Actigraph accelerometry	Longitudinal study, 10 years follow-up	PA fell progressively from 9 years old. Chronological and biological age particularly puberty was identified main factor mostly for girls
14	Bradley et al. (44); USA	801 children	MVPA	Dwelling place	accelerometer	Longitudinal study, 15 years follow-up	PAs declined from the age of 9, due to parental influence and region
15	Francisco et al. (102); Spain	213 children, 105 boys and 108 girls 5–14 years	LPA and MVPA	Gender	ActiGraph accelerometer	Genobox Longitudinal Study	decrease of PAs and an increase of sedentarism in the transition from childhood to adolescence especially for boys (10–11 age)
16	Okazaki et al. (98); Japan	63 Children, 9–14 years	LPAs, MVPAs, SB	Gender	Questionnaire, triaxial accelerometer	Longitudinal study, 5 years follow-up	MVPA, LPA, and SB changed as children transitioned from primary to secondary school (12 years)
17	Nader et al. (45); USA	1,032 subjects, birth-15 years	MVPA	Gender based	Accelerometer	Longitudinal study, 15 years follow-up	PAs started declining at the age of 9 (9–15 years)
18	Wickel and Belton (46); USA	375 Children, 50% boys, birth to 15 years	LPA and MVPA	Gender and BMI based	ActiGraph accelerometers	Longitudinal study, 17 years follow-up	PAs decline from the age of (9–15 years)
19	Pate et al. (99); USA	951 subjects, 10–17 years	PAs (MVPA and Light exercises) except water-based activities	SES, Gender, living area	ActiGraph accelerometers	Longitudinal study, 7 years follow-up	Particular steep decline of PAs was found when children transition from elementary to middle schools (11–13 years old) especially in girls, those who live in rural areas, college education parents.
20	Kemp et al. (50); Australia	3,193 children, 4–5 years,	Non-organized physical activity	Gender based and SES	time-use diaries (TUDs)	Longitudinal study, 9 years follow-up	PA declined at age 11 for boys and 13 for girls, due to Socio-economic position, and screen time (computer use)
21	Rullestad et al. (100); Norway	1,225 subjects, 11–15 years	PAs and Sports	–	Questionnaire	Longitudinal study, 2 years follow-up	Reduction in sports participation during early adolescence (from 13 to 15 years)
22	Boraita et al. (33); Spain	761 subjects, 14 years	N/A	–	Questionnaire,	Cross-sectional study	Social economic level, unfavorable environment, body image dissatisfaction, age, gender, self-esteem, sleep, nutrition.
23	Llorente-Cantarero et al. (65); Spanish	213 subjects, 5–14 years,	LPAs and MVPAs	Gender based, BMI	ActiGraph GT3X+ accelerometers, 6 years follow-up	Cross-sectional 3 years and longitudinal 6years	decrease of PA and an increase of sedentarism in the transition from childhood to adolescence (10, 11), particularly in boys
24	Haas et al. (57)	169 children, 8–12 years	MVPAs	–	Accelerometer	Longitudinal study, 3 years follow-up	A dynamic factor appeared to be enjoyment
25	Eime et al. (59); Australia	440 children, 12–16 years	Leisure-time PAs	Age	Questionnaire	Longitudinal, 3 years follow-up	Perceived barriers: lack of time, energy, and competing priorities.
26	Dishman et al. (58); USA	187 children, 10–15 years		Students who maintained higher self-efficacy and lower perceived barriers		Observational study, 5 years follow-up	Psychological factor: bigger declines in self-efficacy (maintained higher perceptions of barriers to PA, had bigger declines in enjoyment and fitness goals, or (3) had smaller declines in appearance and social goals)
27	Kimm et al. (52); USA	2,379 children, 9/10–18/19 years,	Leisure-time PAs	Racial based (white and black)	Habitual Activity Questionnaire	Longitudinal study, 9 years follow-up	Modifiable: pregnancy, smoking, low level of parental education
28	Francois et al. (103); Canada	781 children, 56% girls aged 10–13 years	Regular physical activities	Gender based	Self-report (checklist)	Longitudinal study, 7 years follow-up	PAs declined due to age (early puberty) for girls. Relative age was associated with boys' PAs decline
29	Portela-Pino et al. (56); Spain	1,081 children, 10/11–14/15 years	Physical activity	Two non-probabilistic, intentional samples	Self-report (Questionnaire)	positivist methodological approach,	PAs decline due to psychological factor (lack of motivation): fatigue and laziness, worrying about body image or feeling anxious in social situations.
30	Dumith et al. (48); Brazil	4,120 subjects, 11–15 years	MVPA	Gender based	Questionnaire	Population-based birth cohort study, 3.5 years follow-up	Predictors to become inactive were higher socio-economic level among boys and increase in screen time among girls.
31	Cairney et al. (61); USA	2,100 subjects, 1,064 boys and 1,036 girls, 11–14 years,	Organized and free play activities	Gender and Age based (biological and chronological)	Participation Questionnaire	Longitudinal study, 4 years follow-up	biological age is stronger predictor of PAs than chronological age and that for organized sport participation
32	Maric et al. (53); Bosnia and Herzegovina	651 children, 327 girls and 324 boys, 14–16 years	PA levels in: Sports, free play, PE	Gender based	Questionnaire for adolescents	Prospective study, 2 years follow-up	Parental education
33	Findlay et al. (49); Canada	8,817 subjects, 4,463 boys and 4354 4–17 years,	Organized PAs	Gender based	Interview (face to face or telephone)	Longitudinal study, 8 years follow-up	PAs decline occurs after preteen years. Associated factors: Household income, parental education, dwelling areas for girls
34	Pabayo et al. (55); USA	889 children, 50.1% boys, birth-15 years	MVPA	Gender based	Actigraph accelerometry	Longitudinal study, 15 years	Area level factors (greater area deprivation) influence lower weekday MVPA for boys.

### 2.5 Quality (risk of bias) assessment

The methodological quality (risk of bias) of the included studies was evaluated using a 10-item quality assessment scale adapted from Van Sluijs et al. (39) (Table 2). The tool was specifically tailored to assess the methodological rigor of studies exploring PA interventions among children and adolescents. For each study, two independent reviewers assessed whether each item on the scale was present or absent. A score of “present” was assigned if the study provided adequate description and compliance with the criterion. If the description was inadequate or missing information the item received an “absent” score. While the “unclear” score was assigned in case of insufficient or ambiguous information, authors were contacted for clarification when necessary. Disagreements were resolved by the lead author through article reassessment. Table 2 provides detailed descriptions of each quality assessment criterion.

**Table 2:** 10-item quality assessment scale.

**Item**	**Description**
1	The criterion for baseline group comparability was considered met if baseline characteristics, such as age and at least one relevant outcome measure, were stratified and presented. For cluster randomized controlled trials and controlled trials, this criterion was satisfied if baseline differences were statistically tested. Additionally, for all study designs, the criterion was considered fulfilled only if any observed baseline differences were appropriately accounted for in the analyses.
2	The randomization process was thoroughly described and implemented in a methodologically sound manner.
3	The unit of analysis was the individual. A negative rating was assigned if the unit of analysis was at the school level or if school-level randomization was not appropriately accounted for in the individual-level analyses.
4	Validated measures of physical activity were utilized. A positive assessment was given if the study reported or referenced the validation of the physical activity measurement tools used.
5	Participant dropout rates were adequately described. A positive rating was assigned if the dropout rate was no greater than 20% for studies with a follow-up duration of 6 months or less, and no more than 30% for studies with follow-up periods exceeding 6 months.
6	The timing of measurements was consistent and comparable between the intervention and control groups.
7	Blinding of outcome assessment was ensured. A positive rating was given if the individuals responsible for measuring physical activity outcomes were blinded to the group allocation of participants.
8	Participants were monitored for a follow-up period of at least 6 months.
9	An intention-to-treat analysis was employed in the study.
10	Potential confounding variables were adequately controlled for in the analyses.

## 3 Results

### 3.1 Age/developmental stage when physical activity levels begin to decline

This systematic review identified 21 studies that reported on the age or developmental stage at which PA levels begin to significantly decline (see Table 1). The findings suggest that this decline may begin as early as age 7 (25).

However, more consistent evidence points to specific ages or developmental stages, with puberty and growth spurts emerging as critical transitional periods. For example, the decline in PA was most frequently reported at age 9, identified in seven studies (40–46), which coincides with the onset of puberty in many children.

Additional studies identified ages 10 and 11 as key moments when PA starts to decline, aligning with the preadolescent phase, a period of biological, psychological, and social change that often leads to reduced PA. For instance, studies by Corder et al. (24) and Kimm et al. (47) suggest that the transition to adolescence, marked by the biological changes of puberty, contributes to a sharp decline in PA. Puberty, particularly in girls, has been associated with an increased sense of body image dissatisfaction, and for both genders, changes in social dynamics and academic pressures play a role in reducing activity levels.

Furthermore, the growth spurt, which typically occurs around ages 10–12, can impact motor skills and coordination, making children feel less confident in participating in physical activities. These physical changes, along with psychosocial factors such as increased academic workloads and shifting social priorities, contribute to the decrease in PA engagement during these key developmental stages.

The consistency of findings across multiple studies underscores preadolescence and the onset of puberty as particularly vulnerable periods for PA decline. Recognizing these critical transition periods is essential for informing the design and timing of interventions aimed at sustaining PA before inactivity becomes entrenched. Therefore, targeting interventions during or just prior to these developmental stages, especially puberty, may be crucial in mitigating the early decline in PA.

### 3.2 Most factors associated with the decline

To better understand the underlying reasons for declining PA levels, this review examined a wide range of individual, social, environmental, psychological, biological, and institutional factors reported in the included studies.

Social factors influencing PA decline included: socioeconomic status (33, 48–51), parental education (49, 52, 53), gender (33, 54), and sports club membership (51). Environmental factors included: an unfavorable environment (33, 49, 51, 55) and country specific differences (54), which may shape accessibility to sports facilities and outdoor activity spaces. Psychological factors play a crucial role, with body image dissatisfaction (33, 54, 56), low self-esteem (33), lack of enjoyment (57), reduced self-efficacy (58), lack of motivation (56), and anxiety (56) all contributing to reduced participation in PA. Individual factors included: lifestyle constraints such as lack of time, energy, and competing priorities (59), smoking (52), fatigue and laziness (56), poor nutrition (33), increased screen time (48, 50), inadequate sleep (33), and active commuting habits (51). Biological factors such as pregnancy (52), early puberty in girls (60), relative age effects in boys (33, 60), and biological/chronological age (43, 61) also influence PA decline. Institutional factors included the reduction of physical education (PE) time and increased academic workload (18), which can significantly limit opportunities for structured PA.

Further analysis categorized these factors into modifiable and non-modifiable influences. A significant proportion of contributors to PA decline are modifiable, meaning they can be addressed through targeted interventions. These include social, psychological, behavioral, and environmental elements, such as socioeconomic status, parental education, sports club membership, body image concerns, self-esteem, motivation, screen time, school workload, and access to PE classes. Since these factors can be shaped by policy changes, school-based programs, and community initiatives, they offer critical opportunities for intervention.

Conversely, non-modifiable factors including gender, country of residence, pregnancy, early puberty in girls, later relative age in boys, and biological/chronological age pose intrinsic challenges that may predispose certain populations to greater declines in PA. Later relative age refers to children born later in the selection year, resulting in a relative age disadvantage compared to their older peers within the same age group, potentially leading to lower physical development and performance in physical activities and sports (62). For instance, early-maturing girls often experience heightened self-consciousness about body image, discouraging participation in PA, while boys with later relative age may struggle with disadvantages in organized sports, reducing engagement. Identifying both modifiable and non-modifiable factors is crucial for designing comprehensive and inclusive intervention strategies aimed at mitigating PA decline across different populations and settings.

## 4 Discussions

### 4.1 Age/stage when physical activity levels begin to decline

This systematic review indicates that PA levels begin to decline as early as age 7, with the most pronounced drop occurring around age 9. This early onset challenges the core objective of PE-to equip individuals with the knowledge, skills, and motivation to engage in lifelong PA (63). The marked decline during the transition from childhood to pre-adolescence reveals a troubling disconnect between educational intent and actual behavioral outcomes. This underscores an urgent need to reevaluate PE strategies and implement targeted interventions that not only maintain PA levels but also foster a sustainable culture of movement beyond school settings.

While PE is a key area for promoting physical activity, it is important to acknowledge that in many Western countries, PE occupies a relatively small proportion of the curriculum. In fact, PE typically accounts for only 2–5% of total curriculum time (23), and this is similarly reflected in children's daily wake time. This limited exposure to structured PA during the school day suggests that reliance on PE alone is insufficient to address the growing trend of physical inactivity. Therefore, it is essential to reconsider the role of PE within the broader context of physical activity promotion, recognizing that effective interventions should extend beyond the classroom to involve families, communities, and broader policy changes that support active lifestyles.

The consequences of this early decline extend beyond reduced PA participation. A sedentary trajectory in these formative years is strongly associated with an increased risk of hypokinetic conditions such as obesity, cardiovascular diseases, and psychosocial challenges, including anxiety and depression. Critically, children who disengage from PA at an early age often find it difficult to re-establish active habits later in life (64). Intervening before inactivity becomes habitual is vital. Consequently, schools, policymakers, and communities should collaborate to integrate movement-rich strategies both within and outside the PE curriculum, ensuring that children remain active throughout their developmental journey.

These findings align with recent studies (65–67), which report declining PA levels and increasing sedentary behavior during the transition to adolescence. However, they diverge from earlier reports (24, 68, 69), which suggested that PA remains stable throughout childhood and only declines during adolescence. Our results also contrast with Kristensen et al. (70), who reported stable PA during the teen years, and differ from prior studies (18, 22) that located the primary decline in adolescence. These discrepancies point to an evolving understanding of PA trajectories and emphasize the need for continued empirical inquiry.

To this end, addressing the early decline in PA is critical for promoting lifelong engagement in PA, enhancing overall health, and improving quality of life. Future research should focus on uncovering the underlying causes of this early drop and developing scalable, evidence-informed strategies to maintain PA across developmental stages.

### 4.2 Factors associated with the decline in physical activity

This review identifies a multifaceted interplay of social, environmental, psychological, biological, individual, and institutional factors that contribute to the decline in PA. Among these, modifiable factors such as self-efficacy, motivation, screen time, and academic workload, emerged as particularly influential. Recognizing these modifiable determinants provides vital entry points for designing effective, targeted interventions.

A noteworthy insight emerging from this finding is the interplay between psychological and behavioral determinants in shaping PA behavior during developmental transitions. While previous studies have highlighted academic pressure (71–73) and social influences that discourage PA (74, 75), this review emphasizes self-efficacy and motivation as pivotal. Strengthening individuals' confidence in their ability to participate in and benefit from PA and nurturing both intrinsic and extrinsic motivation, can serve as protective buffers against declining activity levels.

Furthermore, screen time, a dominant behavioral influence, continues to challenge active lifestyles. While its inverse relationship with PA is well-documented (76–79), this review suggests that interventions should go beyond simply limiting screen exposure. Rather, integrating technology into PA promotion through exergaming, fitness apps, and online activity communities, can help harmonize digital engagement with active behaviors.

Institutional factors also play a critical role. While schools are positioned as key venues for promoting PA (80–82), they may unintentionally contribute to PA decline through increased academic demands and limited PA offerings. These findings reinforce the urgency of restructuring PE to facilitate meaningful, enjoyable participation. Effective strategies may include integrating movement into academic lessons, broadening extracurricular sports options, and fostering strong school-community partnerships.

These insights contribute to the broader literature by affirming the importance of adopting a multidimensional, ecosystems-based approach to PA promotion (83–86). Rather than addressing factors in isolation, future efforts should consider the interplay between social support, environmental access, psychological resilience, and institutional backing to holistically support sustained PA from childhood into adolescence.

#### 4.2.1 Biological and psychosocial maturation: limitations and future directions

Notably, the role of biological and psychosocial maturation in PA decline remains under-investigated in the literature. Despite including search terms related to maturation (physical and psychosocial), this review found that only two of the included studies explicitly examined the effects of biological vs. chronological age on PA trajectories. Cairney et al. (61) observed that when children's ages were aligned by biological maturation (using years to peak height velocity), the apparent influence of chronological age on PA participation diminished (61). In fact, their analysis indicated that biological age was a stronger predictor of PA engagement than chronological age (61). Similarly, Metcalf et al. (43) reported that declines in PA from childhood through adolescence were similarly related to both chronological and biological age. They found that differences in pubertal timing (a key aspect of biological maturation) contributed to the sharper decline observed in adolescent girls (43). These findings suggest that the timing of physical maturation can substantially shape PA behaviors, potentially overshadowing the effects of chronological age alone.

However, the paucity of studies addressing maturation represents a significant limitation in our understanding of why PA declines. The two studies that did consider maturation were conducted in specific contexts and each had limitations. For instance, Cairney et al. (61) focused on self-reported free-play activities among Canadian youth, which may not capture all forms of PA and is susceptible to reporting bias. Metcalf et al. (43) followed a single cohort in one region of the UK, using objective measures (accelerometry) but with a modest sample size that could limit generalizability.

Furthermore, both investigations concentrated on physical maturation (pubertal status and growth markers) and did not explicitly examine psychosocial maturation factors such as evolving identity, motivation, or social roles. These psychosocial changes can shape attitudes and opportunities related to PA as an example, older children often face new academic pressures or social interests that compete with active play, yet none of the studies in our review measured such aspects. This gap highlights the need for caution when interpreting age-related declines in PA: chronological age alone may be an incomplete proxy for the complex developmental changes influencing activity levels.

Looking ahead, future research should prioritize a more nuanced examination of maturation (both physical and psychosocial) in relation to PA behavior. For example, longitudinal studies that incorporate measures of biological maturation for example, age at peak height velocity or Tanner stage alongside chronological age can help clarify how much of the PA decline is due to developmental timing vs. simply getting older. Similarly, adding psychosocial development metrics such as changes in autonomy, self-concept, or peer influence would illuminate how the psychological and social aspects of growing up affect PA trajectories. Emphasizing maturation in study design is crucial for accurately identifying vulnerable periods and tailoring interventions. Strategies to maintain PA might need to be timed around key maturational milestones such as puberty onset and adapted to adolescents' developmental needs. Addressing this currently understudied factor of maturation, researchers and practitioners can better pinpoint when and how to intervene to counteract the decline in PA during youth.

### 4.3 Specific interventions in slowing or preventing the decline

This review highlights a variety of interventions that have proven effective in slowing or preventing PA decline from childhood through adolescence. Consistent with evidence that PA behaviors are modifiable (87), our findings show that the effectiveness of interventions varies depending on their design, implementation, and target population (88). While multicomponent, school-based, theory-informed, and subgroup-targeted interventions emerged as the most successful, a more detailed analysis of the specific components driving effectiveness is essential.

One of the key intervention components identified in this review is PE lesson adaptations. Modified PE lessons, whether standalone interventions or as part of broader programs, were found to significantly increase in-class activity levels. These adaptations often involved increasing intensity for example high-intensity interval training, student-led formats, and content that prioritizes enjoyment. This approach aligns with the finding that interventions which focus on fun, autonomy, and inclusivity in PE are more likely to result in sustained participation (89, 90). The success of these adaptations can be attributed to their ability to engage students more deeply, addressing the common challenge of disengagement during adolescence.

Another highly effective component is family involvement. Studies have shown that interventions incorporating family engagement are particularly successful. For instance, Sutherland et al. (91) and Okely et al. (92) involved families in developing action plans to increase PA. These interventions not only promoted PA within the school environment but also created a supportive home environment that reinforced active lifestyles. Family involvement helps sustain behavioral changes by fostering shared values and social support for PA, particularly in environments where children may lack sufficient external support structures such as in low-income communities. This highlights the importance of a multidimensional approach that involves multiple levels of the ecosystem.

Finally, technology-based engagement has emerged as an increasingly important component in promoting PA, particularly for adolescents. Interventions that incorporated exergaming, fitness apps, and online activity communities have been shown to successfully integrate PA with digital engagement. These interventions, which blend the appeal of digital technology with active behaviors, are particularly effective in bridging the gap for adolescents who may not be as engaged in traditional forms of exercise. The use of pedometers and wearable fitness trackers, as seen in interventions by Lee et al. (93) and Dishman et al. (58), fosters self-monitoring and goal-setting behaviors, contributing to increases in PA by leveraging the self-efficacy and feedback mechanisms inherent in these devices.

Summing up, while school-based and multicomponent interventions were most successful in promoting PA, the specific components such as PE lesson adaptations, family involvement, and technology-based engagement were critical in driving their effectiveness. Moving forward, interventions should prioritize these elements and continue to integrate autonomy-supportive teaching practices and community-level engagement to ensure long-term success in maintaining and increasing PA levels among young people.

It is important to acknowledge a key limitation of the current evidence base. The geographical distribution of the included studies is skewed, with the majority originating from Western, high-income countries. This limits the generalizability of our findings, as the applicability of the identified factors and the effectiveness of the highlighted interventions may vary significantly across different cultural, socioeconomic, and educational contexts. Factors such as societal values toward PA, infrastructure, educational policies, and economic resources can profoundly influence PA trajectories and the success of interventions. Therefore, the conclusion of this review should be interpreted with caution in low- and middle-income countries or in cultures with distinct social norms. This limitation underscores a critical gap in the literature and highlights an urgent need for future research to include more diverse population to build a truly global understanding of PA decline and its solutions.

To this end, this review underscores the critical importance of adopting a comprehensive, multidimensional approach to PA promotion. By integrating school-based initiatives with broader environmental and societal support structures, stakeholders can counteract the decline in PA and cultivate a culture of lifelong PA among young people.

#### 4.3.1 Theory-driven, ecosystem-based interventions in PA promotion

While our systematic review did not explicitly target interventions grounded in ecosystem or whole-system theoretical frameworks, such as Bronfenbrenner's ecological model, we recognize the significant potential of these approaches in addressing the multifactorial nature of PA decline. Ecosystem-based interventions, which consider multiple levels of influence, ranging from individual to community are particularly effective in fostering lasting changes in PA behaviors. Although our review primarily focused on school-based and PE-centered interventions, we noted several relevant studies that employed theory-driven, multilevel approaches but did not meet the inclusion criteria for our review.

One such example is the work by Sutherland et al. (91), who implemented a 2-year, multicomponent intervention guided by a sociological framework. This intervention included seven physical activity strategies such as active recess and sports programs and six strategies aimed at supporting the adoption and implementation of these activities including teacher training and family involvement. The results from this study demonstrated that the intervention successfully increased daily MVPA, with participants averaging approximately seven more minutes of MVPA per day compared to the control group.

Another notable intervention was led by Okely et al. (92), which utilized a community-based participatory research approach combined with an action-learning framework. In this intervention, students, teachers, and community partners collaborated to develop tailored action plans to promote PA, particularly among girls in secondary schools. The intervention successfully improved participants' PA levels and quality of life, showing the impact of a collaborative, community-driven approach.

In Lee et al. (93), a self-efficacy theory-based intervention was applied alongside pedometer use to encourage PA among adolescents. This study found that participants in the intervention group took significantly more steps per day (approximately 467 more steps) than those in the control group. This outcome highlights the importance of integrating behavior-change theories, such as self-efficacy, with simple monitoring tools like pedometers to foster PA engagement in adolescents.

Lastly, Lytle et al. (94) employed a social ecological model to design an intervention aimed at increasing PA among middle-school girls. The intervention included four core components: health education lessons, health promotion activities, physical education teacher training, and school-community partnerships to support PA. The program was highly successful, with a high level of fidelity in its implementation and an increase in after-school PA programs at intervention schools. This study demonstrates the effectiveness of a coordinated, multilevel approach to promoting PA by integrating education, community involvement, and environmental support.

These studies, though not included in our final review, provide valuable evidence supporting the effectiveness of ecosystem-based, theory-driven interventions in addressing PA decline. They highlight the potential of multilevel strategies that involve not only schools but also families, communities, and broader social systems in fostering sustainable physical activity habits. Integrating these kinds of interventions into broader PA promotion efforts could be a crucial step in mitigating the decline in activity levels during childhood and adolescence.

## 5 Future directions

This review underscores promising intervention strategies to address the decline in PA among children and adolescents. However, significant knowledge gaps remain that warrant further exploration to enhance the efficacy, equity, and sustainability of such efforts.

First, many existing interventions lack long-term follow-up, making it difficult to assess the sustainability of behavioral change over time. Future research should prioritize longitudinal designs to evaluate whether PA behaviors are maintained into adulthood and to identify the mechanisms that underpin lasting engagement in PA. Such studies will inform policy-level strategies focused on fostering long-term behavior change, ensuring that interventions do not only yield short-term benefits but lead to sustained improvements in PA.

Second, while targeted interventions particularly those aimed at adolescent girls, have shown potential, broader demographic considerations remain underexplored. Future studies should examine how socio-economic status, cultural context, ethnicity, and geographic location influence PA behaviors. Developing culturally responsive and context-specific strategies will be crucial for improving accessibility and reducing disparities in PA promotion, potentially informing policies that prioritize underrepresented groups and underserved communities.

Third, given the increasing dominance of screen time as a competing behavior, future research should explore the integration of digital tools such as gamification, mobile health applications, and wearable technologies, into PA interventions. These tools may enhance engagement and scalability, particularly when designed with user-centered approaches that align with youths' digital habits. From a policy perspective, recommendations for the incorporation of digital health tools in school curriculums and community programs could ensure a broader reach and greater engagement.

Fourth, school-based interventions often face structural limitations due to rigid curricula and time constraints. Future investigations should explore the complementary roles of after-school programs, family involvement, and community partnerships in promoting sustained PA participation beyond the school setting. Policy reforms should advocate for increased flexibility in school schedules, including expanding PE instructional time and integrating PA into after-school activities and community centers. These reforms would create multifaceted PA opportunities that are accessible and sustainable across different settings.

Fifth, conventional PE curricula tend to prioritize skill acquisition over student engagement. Future research should examine how innovative pedagogical models (PMs) such as Sport Education and Teaching Games for Understanding, can nurture intrinsic motivation, autonomy, and enjoyment, ultimately fostering lifelong PA habits. Policymakers should consider curriculum reforms that emphasize engagement, enjoyment, and autonomy within PE classes, potentially integrating these PMs into national PE standards to ensure that PE curricula are not only skill-focused but also centered around motivational strategies that encourage lasting participation in PA.

Finally, systemic barriers such as limited access to recreational spaces and mounting academic demands continue to inhibit regular PA. Future studies should focus on policy-level solutions that advocate for PA friendly school environments, restructured curricula, and the integration of movement across the school day. Policymakers could prioritize funding for school infrastructure, including the development of outdoor play areas, indoor sports facilities, and safe walking/biking routes. Additionally, academic reforms that allow time for PA during the school day, such as movement breaks and active classrooms, would be crucial in ensuring that PA is seamlessly integrated into daily routines.

Incorporating these policy-level recommendations will be essential for ensuring that interventions not only thrive in experimental settings but are also sustained across population and time, ultimately leading to a culture of lifelong PA.

## 6 Conclusion

This review highlights the concerning early PA decline, beginning as early as age 7 and intensifying around age 9, challenging prevailing assumptions that significant decreases emerge only during adolescence. The trajectory is shaped by a complex interplay of social, psychological, biological, behavioral, and environmental factors, with modifiable determinants such as self-efficacy, motivation, and screen time offering actionable targets for intervention. Among the strategies evaluated, school-based, multicomponent, and theory-informed interventions particularly those that promote autonomy, enjoyment, and social connection, emerged as the most effective in sustaining PA engagement. These findings underscore the urgent need for a paradigm shift in how PA is approached within educational and public health frameworks. In the face of rising global adolescent inactivity, the challenge is clear: how can schools, families, communities, and policymakers collectively reimagine and reinforce systems that prioritize PA? Addressing this issue requires not only targeted strategies but also a broader societal commitment to embedding movement into daily life. Only through coordinated, sustained, and inclusive efforts can we cultivate lifelong PA habits and ensure that PA remains a cornerstone of adolescent development and wellbeing.

## Data Availability

The original contributions presented in the study are included in the article, further inquiries can be directed to the corresponding author.
